# The Cognitive Behavioral Therapy Competence Scale (CCS): initial development and validation

**DOI:** 10.1017/s1754470x21000362

**Published:** 2021-12-15

**Authors:** Natalie Rodriguez-Quintana, Seo Youn Choi, Emily Bilek, Elizabeth Koschmann, Jeffrey Albrecht, Michael Prisbe, Shawna Smith

**Affiliations:** 1 Department of Psychiatry, University of Michigan, North Campus Research Complex Bldg 520, 1600 Huron Parkway, Ann Arbor, MI 48105, USA; 2 Department of Health Management and Policy, University of Michigan, School of Public Health, 1415 Washington Heights, Ann Arbor, MI 48109, USA; 3 Department of Survey Research, University of Michigan, Institute of Social Research, 426 Thompson St, Ann Arbor, MI 48104, USA

**Keywords:** cognitive behavioral therapy, competence, measurement, school-based mental health

## Abstract

**Background:**

Nearly one-third of youth are affected by a mental health disorder, and the majority do not receive adequate care. To improve clinical outcomes among youth, efforts have been made to train providers in evidence-based mental health practices, such as cognitive behavioral therapy (CBT). Such efforts call for valid assessment measures that can inform and evaluate training activities.

**Aims:**

This study presents the development and validation of the CBT Competence Scale (CCS), a brief self-report measure to assess provider competence for CBT delivery.

**Method:**

Participants were 387 school mental health professionals (SMHPs) working with students in Michigan, USA. Initial items (*n*=59) were developed to evaluate competence in delivering common elements of CBT, with competence conceptualized as covering domains of knowledge, perception, and use of CBT techniques. CCS validation proceeded in three steps: using item response theory to select the most important items for assessing knowledge, evaluating the factor structure using exploratory and then confirmatory factor analyses, and examining reliability and validity of the resultant measure.

**Results:**

The validated CCS measure consists of four dimensions of CBT competence across 33 items: Non-behavioral skills, Behavioral skills, Perceptions, and Knowledge. The CCS demonstrated excellent internal consistency and good construct-based validity.

**Conclusions:**

The CCS holds promise as a valid, informative measure of CBT competence appropriate for the school setting, with potential for application in other environments such as mental health clinics.

## Introduction

Nearly one-third of adolescents in the United States will meet diagnostic criteria for a mental health disorder (Merikangas *et al*., 2010). However, of youth who need mental healthcare, only 36% ever receive it (Merikangas *et al*., 2011) and even fewer receive treatment grounded in evidence-based practices (EBPs; Garland *et al*., 2013). Primary barriers to treatment include practical or logistical impediments such as provider waitlists or transportation difficulties, as well as social barriers such as stigma, distrust of clinic-based care, and economic hardship. A significant advantage of school-based delivery of EBPs is the capacity to overcome many of these barriers (Hoover *et al*., 2019; Kern *et al*., 2017).

Cognitive behavioral therapy (CBT) is a leading EBP for addressing symptoms of mental illness, such as depression and anxiety, in school-age youth (Rasing *et al*., 2017; Weisz *et al*., 2017). Its delivery in schools can increase access to, and more equitable delivery of, effective mental health services (Kern *et al*., 2017), and improve students’ clinical and associated educational outcomes (Hoover *et al*., 2019; Joe *et al*., 2009). Recently, studies have reported that about 35–56% of youth who receive mental health services do so in schools (Ali *et al*., 2019; Green *et al*., 2013), underscoring the importance of this delivery setting.

School-based delivery of effective mental healthcare is predicated on school mental health providers (SMHPs), such as social workers, counsellors and school psychologists, having competence in delivering relevant EBPs. Therefore, programmatic efforts to implement CBT in schools must assess and track SMHP’s knowledge, perceptions, and use of CBT to optimize training methods and inform efforts to promote both initial adoption and long-term sustainment. Unfortunately, few validated measures for CBT competence are available (Muse and McManus, 2013; Simons *et al*., 2013), and even fewer are appropriate for school settings. This article reports on the development and validation of the Cognitive Behavioral Therapy Competence Scale (CCS), designed to assess SMHPs implementing CBT with youth.

### CBT competence

A primary factor for ensuring effective and sustainable implementation of CBT interventions is practitioners’ CBT *competence*, defined here as ‘the degree to which a [practitioner] demonstrates the general therapeutic and treatment-specific knowledge and skills required to appropriately deliver CBT interventions’ (Muse and McManus, 2013; p. 485). Competence requires both foundational and procedural skills. Foundational skill refers to factual knowledge of the theory and practice, as well as to practical knowledge of how and when to use that information. Procedural skills are abilities to effectively apply that knowledge in a variety of settings (Miller, 1990; Simons *et al*., 2013). While foundational knowledge of EBPs is necessary for effective and long-term treatment delivery, practitioners must also demonstrate knowledge of when and how to use the skills (Frank *et al*., 2020). Furthermore, motivation to learn, use and apply an EBP is influenced by a provider’s belief that the EBP is likely to be helpful for their target population (Aarons *et al*., 2012; LoCurto *et al*., 2020; Lyon *et al*., 2013; Rogers, 2003). Finally, sustained implementation is predicated on providers initiating appropriate use of relevant treatment skills. Thus, these four criteria together – (a) foundational knowledge, (b) practical or applied knowledge about how and when to use an EBP’s component skills, (c) perceptions of the EBP, and (d) actual use of skills – comprise core competencies that are essential to assess when implementing a new EBP (Miller, 1990; Muse and McManus, 2013).

### Existing CBT competence measures

Two reviews of CBT competence measures have been published within the past decade (Muse and McManus, 2013; Simons *et al*., 2013). Both demonstrate that the majority of existing measures rely on either observational ratings or self-report measures. While relatively objective, observational measurement is burdensome and resource-heavy, as it requires trained experts to observe delivery of at least a subset of clinical skills. The Cognitive Therapy Rating Scale (CTRS; Young and Beck, 1980) is an example of a commonly used observational tool that requires trained raters to observe and code practitioners’ proficiency in specific strategies as well as general therapeutic skills (e.g. agenda-setting and feedback). While observational measures can provide valuable data on a practitioner’s CBT skill, they require a high level of expertise, training and time to conduct, and yet still suffer from poor reliability (Rodriguez-Quintana and Lewis, 2018). Additionally, observational measures fail to examine factors critical for predicting sustained delivery, such as provider perceptions of the EBP (Aarons *et al*., 2012; LoCurto *et al*., 2020). These characteristics render observational tools both impractical and insufficient for use in settings with limited resources and exceptional need, such as schools. Other objective methods for measuring competence include the use of role-plays; however, these too are time-consuming and are probably infeasible for use in school-based implementation efforts (Dorsey *et al*., 2017; Liness *et al*., 2019).

Self-report surveys and knowledge questionnaires are more practical and efficient for implementation purposes. However, use of such tools is less common (Muse and McManus, 2013; Simons *et al*., 2013) due to a number of limitations. First, surveys are frequently designed to accompany a specific CBT protocol or focus on highly technical language and concepts, thus rendering them minimally generalizable (Muse and McManus, 2013; Simons *et al*., 2013). Second, most self-report tools focus exclusively on foundational knowledge, thus failing to measure other competencies known to be critical for implementation success, such as provider perceptions and clinical use of CBT (Herschell *et al*., 2010). One example of a self-report measure of CBT competence that has shown some evidence of validity includes the Cognitive Behavioral Knowledge Quiz (CBT-KQ; Myles and Milne, 2004). The CBT-KQ was developed to evaluate practitioner’s CBT knowledge acquisition after completing a CBT training program. The CBTKQ uses highly technical language and focuses on theoretical knowledge, rather than more practical use, making it most useful for assessing knowledge acquisition among practitioners with extensive and specific training. It is also probably a poor fit for evaluating or informing implementation efforts, as it does not assess foundational knowledge, applied knowledge, perception of skills, or use of skills. Another example is the Knowledge of Evidence Based Services Questionnaire (KEBSQ; Stumpf *et al*., 2009), a measure of knowledge of evidence-based practices in treatments for youth. However, the KEBSQ is considered burdensome, as the reporter has to fill out 160 yes/no decisions, although it has recently been used with 48 yes/no decisions (Okamura *et al*., 2018). Ultimately, while several CBT competence measures are available, none is designed to support or inform implementation in low-resourced settings, such as schools.

### Current study

**Figure 1.**
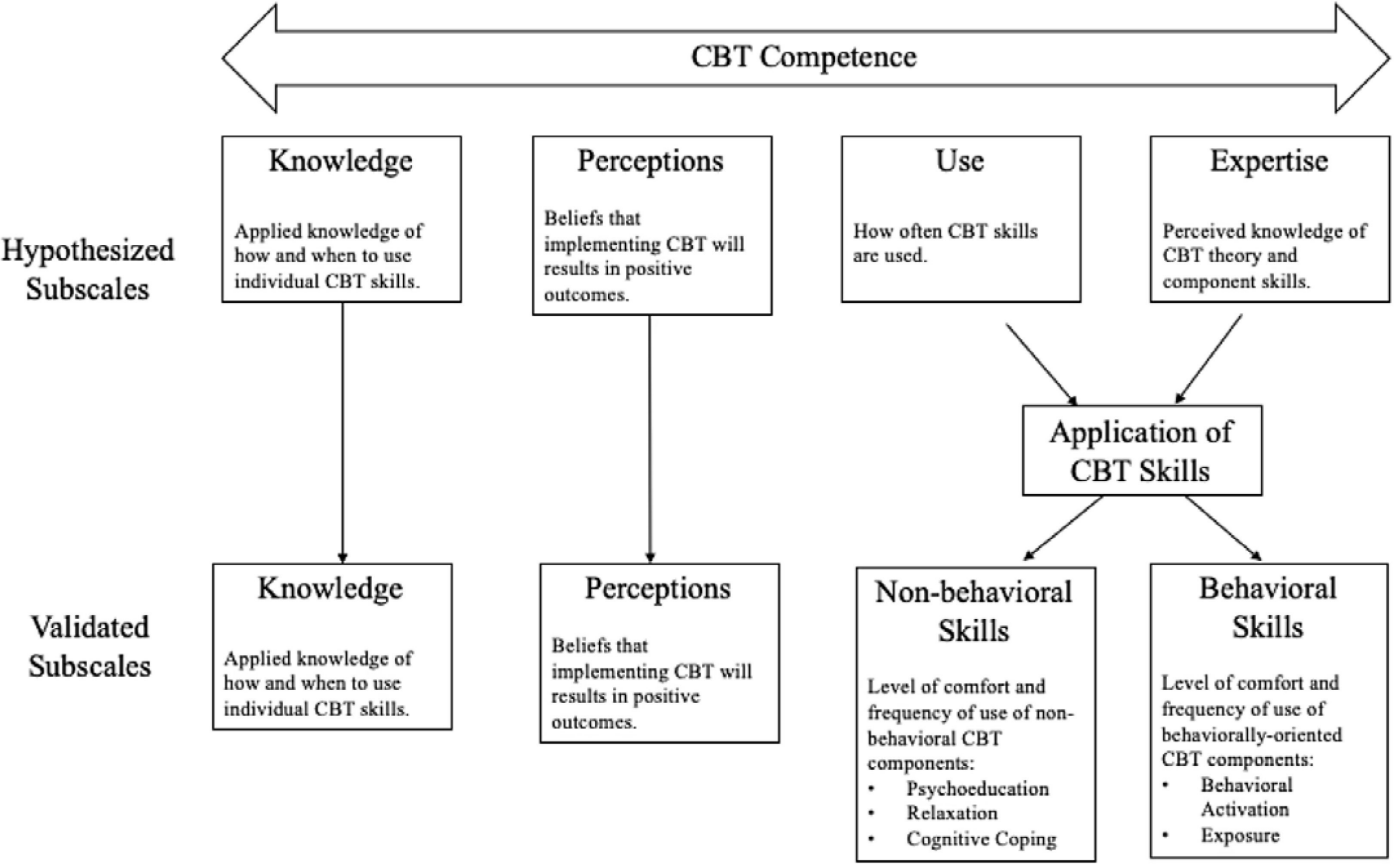
Conceptual model for the CBT Competence Scale (CCS).

The present study sought to address the need for a psychometrically sound measure of competence for implementing CBT in schools. Informed by research identifying key predictors of adoption and sustainment of EBPs, provider competence was conceptualized to include: (a) applied knowledge of how and when to use individual CBT skills (*Knowledge*), (b) perceptions of CBT utility for their local settings (*Perceptions*), (c) frequency of skill use (*Use*), and (d) perceived level of expertise and comfort of CBT theory and component skills (*Expertise*). It was hypothesized that the resultant composite measure, the CBT Competence Scale (CCS) would demonstrate good reliability and validity among school-based providers with various levels of familiarity with CBT including novice clinicians. Figure 1 depicts the hypothesized dimensions for the CCS.

## Method

### Setting

The CCS was originally developed to inform and evaluate training and post-training support offered by the TRAILS (Transforming Research into Practice to Improve the Lives of Students) program, an implementation program for school-based CBT housed at the University of Michigan and currently active in more than 75% of Michigan’s 83 counties. Programming provided to schools by the TRAILS program includes: daylong didactic training in core CBT techniques (e.g. relaxation, cognitive restructuring, exposure), provision of accessible and comprehensive clinical resources and materials, and in-person coaching (for more details on the TRAILS program model, see Koschmann *et al*., 2019 and TRAILStoWellness.org. The TRAILS program model is designed to help overcome primary factors that have been shown to preclude SMHPs from utilizing CBT, including (1) minimal CBT knowledge and skills, (2) lack of access to high-quality training or post-training implementation support, (3) inadequate clinical materials and resources to support direct services to students, and (4) weak perceptions of the utility of CBT for the school setting (Langley *et al*., 2010).

### Procedures

A pool of 59 items was generated by three lead clinicians and CBT experts at the TRAILS program with the goal of covering the most fundamental, common elements of CBT interventions (Chorpita *et al*., 2005). Clinicians helped develop the CBT training of the TRAILS program and their backgrounds are a PhD in Clinical Psychology, a PhD in School Psychology, and a MD with specialization in child psychiatry. Items were reviewed by a national panel of 10 research and clinical CBT experts to establish face validity (i.e. that the items appeared to assess the hypothesized four dimensions of CBT competence) and content validity (i.e. that they covered appropriate content for each domain as it pertains to delivery of CBT in schools). All data collection procedures were approved by the Institutional Review Board at University of Michigan School of Medicine (IRBMED).

### Participants

Participants were 387 SMHPs working with students in Michigan, who completed the CCS assessment through a paper-and-pencil survey or an online survey platform, Qualtrics. Data were collected between September 2018 and February 2019, from three samples: (1) *n*=116 eligible SMHPs recruited to complete the online assessment through a targeted Facebook advertisement (47.7% response rate); (2) *n*=74 SMHPs participating in a countywide TRAILS initiative, who were invited to complete the CCS prior to participating in a daylong CBT training (81% response rate); and (3) *n*=197 SMHPs participating in a large scale implementation trial, the Adaptive School-based Implementation of CBT (ASIC) study (Kilbourne *et al*., 2018), who completed a baseline survey which included the CCS, prior to attending a daylong TRAILS CBT training session (87% response rate). All participants received gift cards (range $10–25) for completing the assessment, which took approximately 25 minutes.

### Measures

#### CBT Competence Scale *(*CCS*)*

The initial CCS had 59 items examining: Knowledge, Perceptions, Use, and Expertise (see Fig. 1 for hypothesized components). The Knowledge items (*n*=23) assessed factual knowledge and practical understanding of CBT by asking SMHPs to identify particular CBT components or select appropriate CBT-based techniques for provided clinical scenarios. For example, one item asked SMHPs to identify the three components of the CBT model (i.e. thoughts, feelings and behaviors); while the other 22 items presented vignettes about six youth experiencing various depression/anxiety symptoms and some brief contextual descriptions, each followed by three to five CBT components from which SMHPs were asked to select the most appropriate for delivery. Expertise items (*n*=12) asked SMHPs to self-evaluate how comfortable they were using common components of CBT, including psychoeducation, relaxation, exposure, behavioral activation, and cognitive coping. The responses ranged from 0 (*Very low: I do not really understand this skill*) to 4 (*Very high: I have significant expertise in this skill and understand it very well*). Use items (*n*=12) queried how frequently SMHPs used these same CBT components with their students on a scale ranging from 0 (*Never: I never or almost never use this skill*) to 4 (*Always: I use this skill all the time with many students*). The Perceptions items (*n*=12) asked SMHPs to rate the effectiveness, appropriateness or helpfulness of CBT: 10 items with response choices of *strongly disagree*, *disagree*, *agree*, *strongly agree*, and *don*’*t know*, one *true/false* item, and one with response choices of *not at all helpful*, *a little bit helpful*, *very helpful*, *extremely helpful*, and *I don*’*t use CBT with students*.

##### Validation measures

###### Prior training and overall confidence.

Two items from the survey were used to assess construct validity: (1) whether SMHPs had any prior graduate-training in CBT and (2) whether they have overall confidence in delivering CBT. We hypothesized that self-reported confidence in CBT would have a moderate correlation with the CCS, and that prior training in CBT would show a weak positive correlation.

###### CBT-KQ and exposure therapy knowledge.

For additional validity assessments, the cohorts recruited through the Facebook advertisement and Washtenaw Intermediate School District (WISD) training (*n*=190) were further asked to complete two sets of additional questions: (1) eight questions from the Cognitive Behavioral Therapy Knowledge Questionnaire (CBT-KQ; Myles and Milne, 2004) that were categorized to the General CBT Issues and Practice of Cognitive Therapy subscales of the CBT-KQ, a 26-item multiple-choice assessment of CBT declarative knowledge, and (2) an open-ended question about exposure therapy: ‘*Imagine you have completed Psychoeducation, Relaxation, and Cognitive Coping with a student/patient with anxiety and at an upcoming session, you will begin Exposure. Identify 4 main components of the session plan that you would prepare.*’ Responses to the exposure therapy question were then graded by a team member with a rubric, and corroborated by two expert CBT practitioners when needed; with SMHPs receiving up to 4 points. At the time the CCS was developed, no comparable instrument measured the intended construct. Therefore, the open-ended exposure question and rubric were generated internally. For both the CBT-KQ and the Exposure Therapy Knowledge, we hypothesized a weak positive correlation with the CCS total and a moderate correlation with the Objective CBT Knowledge subscale, as the CBT-KQ was developed for clinical practitioners focusing on theoretical knowledge.

### Demographics

SMHPs also reported gender, race, ethnicity, highest level of education, current professional position, years in current position, years in professional field, whether they currently saw students for individual or group support/therapy, and whether they had attended professional development on CBT in the previous 5 years.

### Statistical analysis

CCS validation proceeded in three steps. First, in an effort to identify the most important Knowledge items and reduce overall instrument burden, we used item response theory (IRT) to reduce the number of Knowledge vignette cases. In a second step, we combined this shorter set of vignettes with the other remaining 36 items using factor analysis to extract and validate the factor structure of the overall measure. Finally, we assessed the reliability and validity of the final composite scale and subscales: reliability was assessed via Cronbach’s alpha, and validity by examining correlations between the final CCS total and subscales and several previously validated and/or clinically informed measures.

### Item response theory (IRT)

The Knowledge items were shortened using a two-parameter IRT model in Stata 15.1 with full information maximum likelihood (FIML) estimation to handle missing data. IRT provides information about each item and the test as a whole, accounting for the respondent’s ability level (Edelen and Reeve, 2007; Raykov and Marcoulides, 2018; Thomas, 2011; Toland, 2014). The difficulty parameter, *b*, represents the level of ability required to answer each item correctly, and the discrimination parameter, *a*, indicates how well an item differentiates between respondents with similar ability levels. IRT also estimates how much information the instrument captures as a whole^1^, as well as expected test scores at different levels of ability.

Our IRT analyses were applied to 23 graded Knowledge items (0=incorrect or 1=correct). The analysis proceeded as follows: first, we removed items with either a small discrimination parameter (*a*<.5) or an extreme difficulty parameter (*b*>3 or *b*<−3). Second, we selected the combination of vignettes with the highest maximum value of the test information function (TIF), among all possible combinations of a smaller number of cases. Third, similarities between selected items were examined using the items’ information function (IIF). The final set of Knowledge items was selected based on the IIFs and consultation with clinical experts at the University of Michigan.

### Factor analyses

The shortened set of Knowledge items was combined with Perceptions, Expertise, and Use items. Perceptions items were dichotomized for the analyses: ‘1’ indicates positive perceptions and ‘0’ non-positive perceptions.^2^ Factor analyses were used to extract and evaluate the instrument’s factor structure. The sample was randomly split, stratified by recruitment method. The first half (*n*=193) was used for exploratory factor analysis (EFA) and the second (*n*=194) for confirmatory factor analysis (CFA). Expertise items and Use items were considered continuous; Perceptions items and Knowledge items were analysed as binary. Factor analyses were conducted using the fa function in the psych package and cfa function in the lavaan package in R 3.5.2. Missing data were handled using pairwise deletion.^3^

#### Exploratory factor analysis

To account for varying levels of measurement, a correlation matrix combining Pearson, tetrachoric, and biserial correlations was calculated for the EFA. Minimum residual factoring with oblique rotation was used to account for presumed correlation between factors. Items with unique contributions to one factor (i.e. primary loading ≥0.40 and cross-loadings ≤0.30) were retained, and the number of factors retained was determined based on eigenvalues, scree plot, variance explained, and factor loadings (Fabrigar *et al*., 1999; Zwick and Velicer, 1986). Input was also sought from clinical experts throughout the process and used to adjudicate when criteria consensus was not achieved.

#### Confirmatory factor analysis

CFA was used to confirm the EFA factor structure. Parameters were estimated using the weighted least square mean and variance adjusted estimator (WLSMV) and robust standard errors to account for non-continuous items (Li, 2016). Model fit was assessed using the Satorra-Bentler scaling-corrected test statistic (recommended when *n*<250 and the normality of variables is limited; Hu and Bentler, 1999; Yu, 2002), which suggests a good model fit when the Tucker-Lewis index (TLI) and comparative fit index (CFI) exceed .95 and the root mean square error of approximation (RMSEA) index is less than 0.05 (Baldwin, 2019; Medina *et al*., 2009). Two additional measures, chi-square test and the standardized root mean square residual (SRMR) are also reported, but were not used to determine fit, given sensitivity to sample size (Hu and Bentler, 1999).

### Reliability and validity analyses

Cronbach’s alpha was used to test the internal consistency reliability of final CCS total scale and subscales. Construct validity was assessed by examining how CCS total and subscale scores were correlated with four validation measures as discussed above. Our *a priori* hypotheses for the relationship between these measures and the CCS total and subscale score are shown in Table 1.

## Results

### Sample demographics

Participants were predominantly white/Caucasian (87.9%), female (88.1%), and held Master’s degrees in social work, counselling or psychology (84.8%). They had been in their current professional fields for a mean of 12.5 years (range 0–45, standard deviation (*SD*)=9.05, median=11) and in their current positions for a mean of 7.6 years (range=0–45, *SD*=7.58, median=4.25); 50.1% were school counsellors and 29.5% were school social workers; the remainder were other professionals working with students, including school psychologists, behavioral intervention specialists, and school nurses. Almost all of participants (92.8%) saw students for individual counselling, and 49.6% conducted support groups. The majority (62.5%) had attended professional development on CBT in the past 5 years, and 38.8% had received graduate training in CBT.

### Item response theory

Twenty-three graded items of Knowledge were analysed using a two-parameter IRT model. Of 387 respondents, 14 (4%) who had no response on all 23 Knowledge items were excluded from IRT modelling, leaving *n*=373 SMHPs. IIF criteria were used, first, to remove two items with a discrimination parameter (*a*) less than .5 and a difficulty parameter (*b*) greater than 3 or less than −3. The TIF for the resulting 21-item model [a maximum TIF value, I(θ)=4.44 at latent ability level, θ=−.42] was comparable to the TIF from the 23-item model [I(θ)=4.49 at θ=−.42]. Second, the TIFs of 44 models that selected a smaller number of vignettes plus the CBT model question were compared [I(θ) ranged from 1.77 to 3.77] and three models with the highest I(θ) were selected for further examination. Next, these selected models were closely examined in terms of discrimination and difficulty parameters and IIFs in tandem with clinical expert consultation.

The final 15-item model, which includes the CBT model and four vignettes, has I(θ)=3.67 at θ=−.25, with standard error of the ability estimation (SEE)=.522 (see Appendix A in Supplementary material). Above-average respondents were expected to score >=8.3 (out of 15), and 95% of respondents were expected to score 3.17–12.7. Dropping two of six vignette cases reduced the maximum TIF value by 0.8, and the 15-item model provided sufficient amount of information over a broad range of item difficulty, while also substantially reducing respondent time and cognitive burden.

### Factor analyses

Factor analyses were performed with 51 items: the 15 IRT-informed set of Knowledge items and 36 items on Perceptions, Expertise, and Use. Seventy-five (19%) respondents had missing data for one or more item, which were handled using pairwise deletion.

#### Exploratory factor analysis

Nearly all selection criteria endorsed the 4-factor solution; factor loadings for 5- and 6-factor solutions further found that no items had loadings above 0.4 for the final factor(s). An iterative process was then used to retain or remove items based on the statistical and conceptual inclusion criteria described above. In the first iteration, we removed ten items with primary factor loadings <0.4 and four items with cross-loadings >0.3. In the second iteration, two items were removed due to cross loadings >0.3. In the third iteration, one item with a low primary loading was removed. This set of items (*n*=34) was then presented to clinical experts to assess face validity; they suggested removing three items due to ambiguity and adding back two items to ensure proper coverage of key CBT elements. The four factors on *n*=33 final items explained 52% of the total variance (16, 16, 12 and 8%, respectively).

#### Confirmatory factor analysis

CFA with four latent, correlated factors was used to test the identified factor structure.^4^ Standardized factor loadings and fit statistics are presented in Table 2. Goodness-of-fit indices indicated a fair to good fit using the Satorra-Bentler scaling-corrected test statistic.

### Final CCS description

The final scale included 33 items loading uniquely on four factors, defined below. Scoring instructions can be found in Appendix B of the Supplementary material. Notably, two of the originally proposed dimensions, Expertise and Use, did not inform separate factors but were split with respect to non-behavioral and behavioral CBT skills (see Fig. 1 for details).

#### Non-behavioral skills (10 items)

The first factor reflects the degree to which SMHPs report being comfortable with and frequently using foundational, non-behaviorally oriented CBT components (i.e. psychoeducation, relaxation, and cognitive coping). Six items reflect the frequency of SHMP skill usage, and the other four items reflect self-rated expertise in implementing those skills.

#### Behavioral skills (8 items)

The second factor reflects SMHPs’ comfort with and use of two advanced, behaviorally oriented CBT components: behavioral activation and exposure. Four items reflect the frequency of SMHPs skill usage and four reflect self-rated expertise and comfort in implementing those skills.

#### Perceptions (6 items)

The third factor reflects SMHPs’ beliefs that implementing CBT will result in positive outcomes for their students. Six items reflect the level of agreement of SMHPs with statements about CBT’s appropriateness and helpfulness.

#### Knowledge (8 items)

The fourth factor captures SMHPs’ applied knowledge and appropriate use of common CBT components. Eight items reflect the SMHP’s knowledge about appropriate usage of CBT skills in a set of case vignettes and the components of the CBT model.

### Reliability and validity

Summary statistics and correlations for the total scale and the four subscale scores are shown in Table 3.^5^ The Behavioral Skills subscale showed a lower mean score (0.84) while the Perceptions subscale had a higher mean score (3.07). The four factors were weakly-to-moderately correlated, with the highest correlation between the two skills (*r*=0.57). These factors were weakly correlated with Perceptions (*r*=0.34 and 0.26, respectively). Knowledge had the weakest correlation with other three factors (*r*=0.20, 0.01 and 0.23, respectively) Cronbach’s alpha internal consistency for total CCS score was 0.92, and subscale scores were 0.94, 0.92, 0.77 and 0.58, respectively. The Knowledge subscale was retained for the final CCS scale, in spite of its relatively lower level of internal consistency, as clinical experts believed it reflected an important, distinct construct for understanding and assessing CBT practice among non-clinicians. Notably, several other scales measuring cognitive constructs or knowledge assessments also report Cronbach’s alpha below .60 (Taber, 2018).

Table 4 summarizes correlations between CCS total and subscales scores and four validation measures. As hypothesized, CCS total score showed weak positive correlations with CBT-KQ and Exposure Therapy Knowledge (*r*=.04, *p*=0.585 and *r*=.18, *p*=.02, respectively); the Objective CBT Knowledge subscale had moderate correlations with these measures (*r*=.31, *p*<.001 and *r*=.27, *p* <.001, respectively), among the cohorts recruited through Facebook advertisement and WISD training (*n*=190). CCS total score was positively correlated with prior graduate training in CBT (*r*_pb_=.27, *p*<.001), and with overall confidence in CBT (*r*_pb_=.35, *p*<.001), among all participants (*n*=387). CCS total scores were also significantly higher among those with confidence in using CBT (*t*_356_=7.11, *p*<.001) and those with graduate training in CBT (*t*_362_=5.41, *p*<.001). All CCS subscales except for Knowledge showed similar relationships.

## Discussion

The aim of this manuscript was to develop and validate a multi-dimensional measure of CBT competence for use with providers in school or other similar settings. Overall, following established criteria (Lewis *et al*., 2015; Lewis *et al*., 2018) the CCS demonstrated adequate psychometric properties in a sample of SMHPs, including excellent internal consistency of the total scale (Cronbach’s α=0.92), good convergent validity, excellent known-groups construct validity, and good dimensionality/structural validity, with factors explaining more than 50% of variance and a fair-to-good CFA model fit.

The CCS is a psychometrically sound and innovative multi-dimensional measure of CBT competency: Knowledge, Perceptions, Non-behavioral Skills, and Behavioral Skills. The CCS measure is a lower burden than other approaches, taking approximately 10 minutes for most respondents to complete. Practically, the CCS can be used at the total score, subscale scores, and/or item-by-item to identify specific implementation targets. For example, utilizing dimension scores might be particularly helpful in the Non-behavioral and Behavioral Skills subscales, as they encompass both frequency and comfort in using individual skills. For example, low usage and frequency of use of exposure might lead to follow-up training on this specific skill.

The CCS differentiated between SMHPs’ comfort with (as defined by both their perceived expertise and reported frequency of use of) non-behavioral and behavioral CBT common elements (i.e. psychoeducation, relaxation, and cognitive coping versus behavioral activation and exposure therapy). These findings are consistent with research indicating that behavioral elements are often under-implemented (Whiteside *et al*., 2016; Wolk *et al*., 2019), although these components, in addition to cognitive restructuring, are potentially the most effective components for youth experiencing depression or anxiety (e.g. Oud *et al*., 2019; Whiteside *et al*., 2020). Indeed, SMHPs scored the lowest on the Behavioral Skills subscale. By differentiating between these two types of skill, CBT training programs can use the CCS to identify SMHPs for whom further scaffolding and training is needed to use the more advanced and most effective CBT components.

The relatively higher mean score for the Perceptions subscale might indicate that most SMHPs hold positive views on CBT in general, regardless of their comfort with using particular elements with students. Another explanation could be that participants were self-selected for the training, and might have come in with higher perceptions about CBT than the average SMHP. Prior research has indicated that positive perception is associated with sustained use over time (LoCurto *et al*., 2020). The Perceptions subscale was weakly to moderately correlated with other three subscales. In addition, the Knowledge subscale was weakly correlated with the other three CCS subscales. Particularly, SMHPs with higher scores on Knowledge were more likely to report slightly higher frequency of use and level of comfort using non-behavioral skills, but no such correlation was found with behavioral skills. These findings might reflect that knowledge is a precursor of skill use (Jensen-Doss *et al*., 2008).

The absent correlation between the Behavioral Skills and Knowledge subscales was especially notable, contradicting expectations of their positive correlation given that literature points to knowledge preceding and being necessary for practice (Nelson and Steele, 2007; Rogers, 2003). There are several possible interpretations. First, as noted previously, few SMHPs reported high scores on the Behavioral Skills scale in our sample. Perhaps the absent correlation reflects a restricted range, thereby limiting our ability to examine scale correlates. It is also possible that the Knowledge subscale measures a broad range of knowledge related to CBT, which takes time to achieve as skills develop and generalize. To that end, respondents who had attended CBT training in the past 5 years demonstrated greater internal consistency (Cronbach’s α=0.66, *n*=232) than those who had not (Cronbach’s α=0.40, *n*=134). Future studies may examine if internal consistency for the Knowledge subscale, as well as the correlation between the Behavioral Skills and Knowledge subscales, increases over time as SMHPs receive CBT training or implementation support. Additionally, concurrent evaluation of implementation environment may help document implementation barriers that are external to the provider, such as setting constraints or low administrator support, that would not be measured by the CCS.

Finally, it is possible that SMHPs who reported that they used or felt comfortable using Behavioral Skills may not have accurate knowledge about what these skills entail (Mathieson *et al*., 2010) and may actually be more likely to over-estimate their expertise (Kruger and Dunning, 1999). Research also suggests that knowledge does not necessarily predict use of skills even though it is an important implementation target (Becker-Haimes *et al*., 2017). This discordance between Knowledge and Behavioral Skills subscales underlines the potential benefit of offering a multi-method assessment, such as the CCS that separately measures an individual’s perceived use/expertise with skills and a more objective measure of knowledge. Future research might examine how training and implementation support could be tailored to an individual’s CCS profile.

### Limitations

This study has several additional limitations. First, SMHPs were not sampled randomly, but rather were included through either self-selection into a CBT training program or opting to complete a survey made available through targeted social media advertisements. Further validation efforts will need to explore the generalizability of the CCS, including evaluations of whether the observed psychometric properties of the CCS replicate with more representative samples, other populations (e.g. expert clinicians), or in different settings (e.g. in low- versus high-resource schools). Second, the size of the sample used for the analyses was only moderate and further validation with a larger sample would be beneficial. Although there is no consensus about the sample size requirement for a factor analysis or for a two-parameter IRT model, studies recommend sample sizes greater than 500 for more accurate estimations (Boateng *et al*., 2018). Third, predictive validity could not be evaluated in this study; future studies should assess the relationship between the CCS and key outcomes, such as CBT adoption. Fourth, even though the CCS is a lower burden measure of CBT competency, as compared with other approaches such as observational coding, it is still 33 items and takes an average of approximately 10 minutes to complete. This is probably still burdensome, especially for providers that have high volumes of cases, such as in schools that are under-resourced. More work should continue to be done to develop more pragmatic and lower burden competency measures that reduce barriers to use. Fourth, there is lack of comparison to the gold-standard approach to evaluating competency, direct observational coding. The CCS is a self-reported measure of competency, and prior literature suggests that practitioners might over-estimate their skills in self-reported measures (Hogue *et al*., 2015). Fifth, the CCS focuses primarily on a specific set of skills used to treat depression or anxiety, which means it is not exhaustive and future iterations of the measure should consider additional skills and presenting problems.

### Conclusion

The CCS is a pragmatic tool that can be utilized by a variety of stakeholders in need of evaluating SMHPs’ competence in delivering common CBT-based intervention components. The tool also has potential to serve as a CBT competence tool for broader settings undergoing implementation efforts. As demand for school- and community-based mental health EBPs increases, brief and reliable measures of SMHPs’ CBT competence will be needed to evaluate baseline skill and readiness for CBT delivery. The CCS holds promise for supporting the implementation of CBT skills among high-need settings, such as schools.

## Supplementary Material

1

## Figures and Tables

**Table 1.** *A priori* hypothesized relationships between CCS and validation measures

	CCS total	Non-behavioral skills	Behavioral skills	Perceptions	Knowledge

**CBT-KQ**	(+) Weak	(+) Weak	(+) Weak	(+) Weak	(+) Moderate
**Exposure therapy Knowledge**	(+) Weak	(+) Weak	(+) Weak	(+) Weak	(+) Moderate
**graduate training**	(+) Weak	(+) Weak	(+) Weak	(+) Weak	(+) Weak
**Confidence in CBT**	(+) Moderate	(+) Moderate	(+) Moderate	(+) Moderate	(+) Moderate

CBT-KQ, Cognitive Behavioral Therapy Knowledge Questionnaire; (+), positive correlations.

**Table 2.** Factor loadings from exploratory factor analysis (EFA) and confirmatory factor analysis (CFA)

	EFA factor loadings				CFA factor loadings
	1	2	3	4	

**Non-behavioral CBT skills**					
Frequency: Teach relaxation	**0.80**	−0.04	−0.17	−0.02	0.57
Frequency: Teach to challenge automatic thoughts	**0.76**	0.01	0.13	0.08	0.87
Frequency: Teach to identify coping thoughts	**0.76**	0.13	0.02	0.04	0.81
Frequency: Teach to identify thoughts, emotion, behavior in a given situation	**0.72**	−0.06	0.09	−0.11	0.63
Frequency: Teach to recognize automatic thoughts	**0.64**	0.20	0.15	−0.06	0.79
Expertise: Teach relaxation	**0.58**	0.07	0.01	0.10	0.59
Expertise: Teach to identify coping thoughts	**0.56**	0.18	0.13	0.14	0.67
Frequency: Provide age-appropriate psychoeducation	**0.54**	0.16	0.04	0.10	0.65
Expertise: Teach to challenge automatic thoughts	**0.51**	0.07	0.28	0.09	0.78
Expertise: Teach to identify thoughts, emotion, behavior in a given situation	**0.47**	0.09	0.20	0.02	0.54
**Behavioral CBT skills**					
Expertise: Assist in behavioral activation planning	0.01	**0.81**	0.02	−0.09	0.76
Expertise: Implement exposure plan	−0.06	**0.79**	0.17	0.05	0.80
Frequency: Implement exposure plan	−0.02	**0.78**	−0.04	−0.13	0.66
Frequency: Assist in fear hierarchy development	0.12	**0.77**	−0.21	0.01	0.70
Expertise: Provide rationale for exposure	0.02	**0.77**	0.13	0.18	0.93
Expertise: Assist in fear hierarchy development	−0.03	**0.75**	0.07	0.17	0.88
Frequency: Provide rationale for exposure	0.23	**0.71**	−0.06	−0.02	0.88
Frequency: Assist in behavioral activation planning	0.10	**0.71**	−0.07	−0.16	0.64
**CBT perceptions**					
Perception: CBT improves clinical outcomes	0.22	0.01	**0.82**	−0.11	0.79
Perception: CBT appropriate for diverse students	−0.03	−0.12	**0.82**	−0.03	0.85
Perception: CBT too complicated (R)	−0.11	0.11	**0.78**	0.20	0.79
Perception: CBT not appropriate in real world (R)	0.07	−0.06	**0.74**	0.22	0.73
Perception: CBT effective for children exposed to trauma	0.12	0.08	**0.72**	−0.17	0.75
Perception: CBT appropriate for children with severe symptoms	0.05	0.21	**0.60**	−0.11	0.74
**Objective CBT knowledge**					
Damien: Exposure selection	−0.02	0.09	−0.03	**0.74**	0.47
Anthony: Psychoeducation – feelings	0.11	−0.04	0.03	**0.56**	0.24
Jessica: Relaxation	−0.22	0.09	0.12	**0.54**	0.13
Anthony: Psychoeducation – rationale	0.22	−0.07	−0.09	**0.54**	0.50
Dana: Exposure selection	0.12	−0.10	0.02	**0.51**	0.53
Damien: Cycle of avoidance	0.09	−0.09	0.12	**0.46**	0.22
Anthony: Behavioral activation	0.26	−0.16	−0.14	**0.45**	0.41
CBT diagram	0.17	0.02	0.19	**0.31**	0.82
Dana: Exposure mechanics	−0.05	0.14	−0.01	**0.21**	0.49

*n*=193 for EFA; the EFA factor loadings are in bold font where an item loads on to the factor (i.e. subscale). *n*=194 for CFA; the CFA factor loadings are standardized and *p*-values are 0.000, except for three items in Objective Knowledge (Anthony: Psychoeducation – feelings (*p*=0.059), Jessica: Relaxation (*p*=0.316), and Damien: Cycle of avoidance (*p*=0.088)). Goodness of fit indices from CFA: *χ*^2^ (d.f.)=804.06, *p*<0.001; CFI=.97; TLI=0.96; RMSEA=0.05, 90% CI [0.04, 0.05]; and SRMR=0.10

**Table 3.** Summary statistics and correlations for the CCS total and subscale scores

							Correlations			
	Mean	SD	Minimum	Maximum	Obs.	Alpha	1	2	3	4

**CCS total**	2.04	0.62	0	3.62	368	0.92	0.73	0.64	0.76	0.56
**CCS subscales**										
**1. Non-behavioral skills**	2.13	0.74	0	4.00	340	0.92	1.00			
**2. Behavioral skills**	0.84	0.81	0	3.88	372	0.94	0.57	1.00		
**3. Perceptions**	3.07	1.14	0	4.00	385	0.77	0.34	0.26	1.00	
**4. Knowledge**	2.10	0.92	0	4.00	366	0.58	0.20	0.01	0.23	1.00

See scoring instructions for details (Appendix B in Supplementary material). Perceptions and Knowledge subscales consist of all dichotomous items; their scores were multiplied by 4 to range from 0 to 4 to compute the total score. Subscale scores were not calculated if a respondent was missing more than half of the subscale items; total score was not calculated if a respondent was missing two or more subscale scores. Cronbach’s alpha of 0.92 for total score was calculated using 33 items in their original scale.

**Table 4.** Construct-based validity evidence correlations

	CCS total	Non-behavioral skills	Behavioral skills	Perceptions	Knowledge

CBT-KQ	0.04 [−0.11, 0.19]	−0.11 [−0.26, 0.06]	−0.09 [−0.24, 0.06]	0.00 [−0.14, 0.15]	0.31*** **[0.17, 0.44]**
Exposure therapy knowledge	0.18* **[0.03, 0.32]**	0.07 [−0.09, 0.23]	0.07 [−0.08, 0.21]	0.08 [−0.06, 0.22]	0.27*** **[0.13, 0.40]**
Graduate training	0.27*** **[0.18, 0.37]**	0.25*** **[0.14, 0.34]**	0.28*** **[0.18, 0.37]**	0.14** **[0.04, 0.24]**	0.08 [−0.03, 0.18]
Confidence in CBT	0.35*** **[0.26, 0.44]**	0.32*** **[0.22,0.42]**	0.41*** **[0.32, 0.49]**	0.17** **[0.07, 0.26]**	0.07 [−0.03, 0.17]

*** *p*<.001

** *p*<.01

* *p*<.05.

95% CIs are in square parentheses. *n*=147–190 for CBT-KQ (a composite score from the graded eight items of CBT-KQ, ranged from 0 to 6), Exposure Therapy Knowledge (range from 0 to 4). CBT-KQ and Exposure Therapy Knowledge were asked only to the two cohorts, those who were recruited through Facebook advertisement and through WIDS training. *n*=329–381 for Confidence in CBT (*vs* none) and Graduate training (*vs* none). Pearson or bi-serial correlations were estimated using the cor.test function in R.

## Data Availability

Data are available on reasonable request to the authors.

## Key learning aims

1. To provide an overview of the importance of measuring CBT competency.
2. To recognize the challenges entailed in measuring CBT competency in under-resourced settings.
3. To understand the development and validation of the CCS measure.

## Key practice points

1. Valid, reliable, and pragmatic tools of CBT competency are needed for routine care settings. The CCS attempts to fill this gap.
2. Assessment of the CCS in school-based settings indicates that it has adequate psychometric properties.
3. It will be important to evaluate the psychometric properties of the CCS in other routine care settings.

## Footnotes

1 Test information function (TIF) shows how much information the instrument captures as a whole, as a function of the latent ability (θ), or the sum of item information functions (IIFs): where *j* is the number of items in the test. The item information functions (IIF) shows how much empirical information each item is providing to the instrument at an ability level of θ: where is the probability of correct response given the ability level θ, or 1/[1_exp(−)], is the item discrimination parameter, and is the item difficulty parameter.

2 The ten items were dichotomized to ‘1’ if the response was strongly agree/agree, and ‘0’ otherwise. The true/false question was coded as ‘1’ if true and ‘0’ if false. The last item was dichotomized to ‘1’ if very helpful/extremely helpful, and ‘0’ otherwise.

3 Among the 387 respondents, 75 cases (19%) had missing information. Among those with missing data, 11 (15%) had missing information on one item, 45 (60%) had missing on 2–10 items, and the remaining 19 (25%) had more than 10 items missing.

4 Residual variances between the items for Perceived CBT Expertise and Frequency of CBT Use that reference the same CBT component (e.g. individuals reporting on their expertise and frequency of use of psychoeducation) were also specified as correlated.

5 The subscale scores were not calculated if more than half of the subscale items were missing: 34 cases for Non-behavioral Skills, 1 case for Behavioral Skills, 0 cases for Perceptions, and 7 cases for Knowledge. The total score was not calculated if two or more subscales were missing values (19 cases). Note also that the Perceptions and Knowledge subscales are comprised of binary items and thus their subscale scores were multiplied by 4 to normalize the range from 0 to 4 for all subscales.
