# Supplementary material for: The Cognitive Behavioral Therapy Competence Scale (CCS): initial development and validation
Source: Cogn Behav Therap. Author manuscript; Available in PMC 2022 Jul 22. (PMC9307077; doi:10.1017/s1754470x21000362)
Supplement: 1 [file NIHMS1759968-supplement-1.docx]

**Appendix A**

Table S1. Comparisons of three IRT models

|  | **The maximum value from the TIF at theta, I(θ)** | **SEE(θ)** | **Expected range of scores for 95% of respondents**  **(The maximum possible score)** | **Expected score for**  **above-average respondents** |
| --- | --- | --- | --- | --- |
| **23-item model** | 4.487 (θ=-.415) | .472 | 4.95-18.10 (23) | 12.0 |
| **21-item model** | 4.443 (θ=-.415) | .474 | 4.8-17.50 (21) | 11.7 |
| **15-item model** | 3.674 (θ=-.254) | .522 | 3.17-12.70 (15) | 8.3 |

*Note.* The results from a two parameter IRT. 23-item model includes all objective knowledge measures from six vignettes; 21-item model shows when items with a small discrimination parameter and/or extreme values of difficulty parameter were removed; 15-item model is the finally selected one including items from four vignettes. TIF: Test Information Function. SEE: Standard error of the (ability) estimation. Expected scores are based on Test Characteristic Curves. θ = latent ability level

Figure S1. Test Information Function (TIF)

Note: The TIFs from the IRT-2PL models. 23-item model includes all objective knowledge measures from six vignettes; 21-item model shows when items with a small discrimination parameter and/or extreme values of difficulty parameter were removed; 15-item model is the finally selected one including items from four vignettes.

**Appendix B**

**CBT Competence Scale (CCS)**

Please indicate your **level of expertise** for each strategy listed using the following 0-4 scale:

***0 = Very low****: I do not really understand this skill*

***1 = Low****: My understanding of this skill is limited*

***2 = Moderate****: I think I understand this skill, but I would benefit from additional practice/learning*

***3 = High****: I understand this skill well*

***4 = Very high****: I have significant expertise in this skill and understand it very well*

| Level of expertise: | 0  Very Low | 1  Low | 2  Moderate | 3  High | 4  Very High |
| --- | --- | --- | --- | --- | --- |
|  | | | | | |
| 1. Teaching students to notice and measure their thoughts, emotions, and behavior in the context of a given situation |  |  |  |  |  |
| 1. Teaching 3-5 different relaxation or physical coping techniques |  |  |  |  |  |
| 1. Teaching students to challenge their unhelpful or illogical automatic thoughts |  |  |  |  |  |
| 1. Teaching students how to replace automatic thoughts with more helpful or realistic “coping” thoughts |  |  |  |  |  |
| 1. Providing a definition and rationale for exposure |  |  |  |  |  |
| 1. Helping students to design and build a Fear Hierarchy or Fear Ladder |  |  |  |  |  |
| 1. Implementing an exposure plan and incorporating incentives |  |  |  |  |  |
| 1. Creating and using a Behavioral Activation Schedule |  |  |  |  |  |

Please indicate **how often** you use each strategy listed when working with students with depression and/or anxiety:

***0 = Never****: I never or almost never use this skill*

***1 = Rarely****: I rarely use this skill*

***2 = Sometimes****: I use this skill some of the time*

***3 = Often****: I use this skill often*

***4 = Always****: I use this skill all the time with many students*

| Frequency of use: | 0  Never | 1  Rarely | 2  Sometimes | 3  Often | 4  Always |
| --- | --- | --- | --- | --- | --- |
|  | | | | | |
| 1. Providing age appropriate psychoeducation about depression and anxiety |  |  |  |  |  |
| 1. Teaching students to notice and measure their thoughts, emotions, and behavior in the context of a given situation |  |  |  |  |  |
| 1. Teaching 3-5 different relaxation or physical coping techniques |  |  |  |  |  |
| 1. Teaching students to recognize their automatic thoughts |  |  |  |  |  |
| 1. Teaching students to challenge their unhelpful or illogical automatic thoughts |  |  |  |  |  |
| 1. Teaching students how to replace automatic thoughts with more helpful or realistic “coping” thoughts |  |  |  |  |  |
| 1. Providing a definition and rationale for exposure |  |  |  |  |  |
| 1. Helping students to design and build a Fear Hierarchy or Fear Ladder |  |  |  |  |  |
| 1. Implementing an exposure plan and incorporating incentives |  |  |  |  |  |
| 1. Creating and using a Behavioral Activation Schedule |  |  |  |  |  |

For each item, please indicate how much you agree with the statement:

1 = Disagree

2 = Agree

3 = Don’t know

| Perceptions: | 1  Disagree | 2  Agree | 3  Don’t Know |
| --- | --- | --- | --- |
|  |  |  |  |
| 1. Using CBT for the treatment of mental health difficulties in children with exposure to severe trauma (e.g., domestic violence, assault) is an effective approach |  |  |  |
| 1. CBT, if used appropriately, will improve the average clinical outcomes of most students |  |  |  |
| 1. CBT may be appropriate for research clients but not the “real-world” students at my school |  |  |  |
| 1. CBT is appropriate even for students with severe symptoms |  |  |  |
| 1. CBT is too complicated for most students to understand |  |  |  |
| 1. CBT is appropriate for students of a wide variety of ethnicities, cultures, and socio-economic backgrounds |  |  |  |

1. Fill in the blanks on this CBT diagram:

***
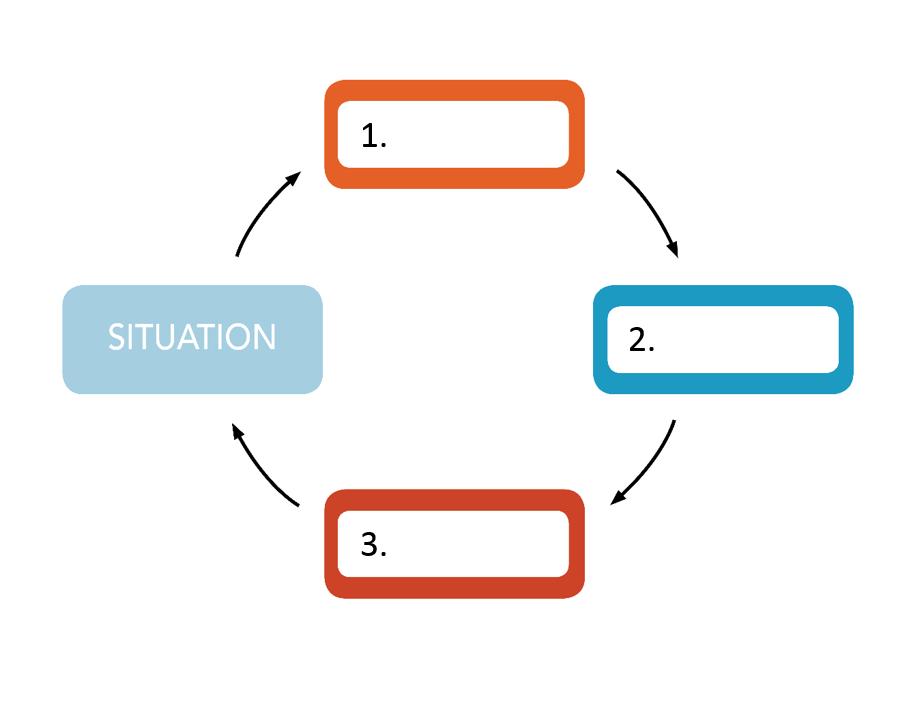
***

**1: ___________________**

**2: ___________________**

**3: ___________________**

You will now read a series of brief case vignettes. Please answer each question about each vignette by choosing the best answer from a CBT perspective.

***Damien is a 14 year old boy with social anxiety. He has trouble speaking up in class and has stayed home sick for his last 3 class presentations. Please answer the following questions about Damien.***

1. One exposure that you might have Damien do would be:
2. Practice his next presentation until he feels confident
3. Have him practice relaxation right before his next presentation
4. Teach him about the cycle of avoidance
5. Ask him to raise his hand in class
6. It's important to teach Damien about the cycle of avoidance. This would include helping him understand that:
7. He will learn coping skills through avoidance
8. Avoidance feels good in the short term
9. He will need to learn when to use avoidance
10. Avoidance is more common in children and teens than in adults

***Dana is a 16 year old girl with generalized anxiety disorder and depression. She worries a lot about her schoolwork, tends to be very perfectionistic, and has recently quit the basketball team to spend more time doing homework and studying for tests. Please answer the following questions about Dana.***

1. Dana should feel anxious during her exposures.
2. True
3. False
4. An appropriate target for exposure for Dana might be:
5. Starting her homework early
6. Offering to give a presentation at school
7. Making an intentional mistake on her homework
8. Playing basketball with friends

***Anthony is a 12 year old boy who has been diagnosed with depression. He doesn’t engage much with peers or friends and has stopped participating in any after school activities. He reports that he spends a lot of time watching videos and has a hard time sleeping. Please answer the following questions about Anthony.***

1. The most effective treatment component for Anthony would likely be:
2. Active listening
3. Behavioral activation
4. Relaxation therapy
5. Exposure
6. A "feelings thermometer" would help Anthony learn that:
7. Feeling down is different from feeling devastated
8. His emotional health is directly linked to physical health
9. His depression makes him more vulnerable to getting sick
10. Feelings can be "hot" or "cold"
11. According to CBT, providing psychoeducation to clients like Anthony is important because it helps to:
12. Determine the root cause of his symptoms
13. Normalize his symptoms and engage him in treatment
14. Provide content to explore in his treatment
15. Teach about mental illness

***Jessica is a 17 year old girl with depression. She has stopped spending time with her friends and family, and spends most weekends in her room with the door closed. Please answer the following questions about Jessica.***

1. Relaxation may be important for Jessica because:
2. Jessica has some anxiety symptoms that would respond well to relaxation
3. Jessica's depression is too severe for behavioral activation, so she needs to learn relaxation first
4. Relaxation is a core CBT component, and she will not improve without learning all the skills
5. Relaxation may help with sleep difficulties that are common in depression

**CBT Competence Scale (CCS) Scoring Instruction**

Abbreviated Items, Factor Loadings, Cronbach’s Alphas, and Scoring

The CCS assesses the degree to which a non-clinical expert is comfortable with foundational, cognitive CBT components and with more intensive, behavioral CBT components, perceives of CBT, and is knowledgeable of CBT theory and clinical application.

| **Item #** | **Abbreviated Items** | **EFA**  **Factor**  **Loading** | **CFA**  **Factor**  **Loading** | **Alpha** |
| --- | --- | --- | --- | --- |
|  | **Scale 1: Non-behavioral Skills** |  |  | 0.92 |
| 1 | Expertise: Teach to identify thoughts, emotion, behavior in a given situation | 0.47 | .54 |  |
| 2 | Expertise: Teach relaxation | 0.58 | .59 |  |
| 3 | Expertise: Teach to challenge automatic thoughts | 0.51 | .78 |  |
| 4 | Expertise: Teach to identify coping thoughts | 0.56 | .67 |  |
| 9 | Frequency: Provide age appropriate psychoeducation | 0.54 | .65 |  |
| 10 | Frequency: Teach to identify thoughts, emotion, behavior in a given situation | 0.72 | .63 |  |
| 11 | Frequency: Teach relaxation | 0.80 | .57 |  |
| 12 | Frequency: Teach to recognize automatic thoughts | 0.64 | .79 |  |
| 13 | Frequency: Teach to challenge automatic thoughts | 0.76 | .87 |  |
| 14 | Frequency: Teach to identify coping thoughts | 0.76 | .81 |  |
|  | **Scale 2: Behavioral Skills** |  |  | 0.94 |
| 5 | Expertise: Provide rationale for exposure | 0.77 | .93 |  |
| 6 | Expertise: Assist in fear hierarchy development | 0.75 | .88 |  |
| 7 | Expertise: Implement exposure plan | 0.79 | .80 |  |
| 8 | Expertise: Assist in Behavioral Activation planning | 0.81 | .76 |  |
| 15 | Frequency: Provide rationale for exposure | 0.71 | .88 |  |
| 16 | Frequency: Assist in fear hierarchy development | 0.77 | .70 |  |
| 17 | Frequency: Implement exposure plan | 0.78 | .66 |  |
| 18 | Frequency: Assist in Behavioral Activation planning | 0.71 | .64 |  |
|  | **Scale 3: Perceptions** |  |  | 0.77 |
| 19 | Perception: CBT effective for children exposed to trauma | 0.72 | 0.75 |  |
| 20 | Perception: CBT improves clinical outcomes | 0.82 | 0.79 |  |
| 21 | Perception: CBT not appropriate in real world (R) | 0.74 | 0.73 |  |
| 22 | Perception: CBT appropriate for children with severe symptoms | 0.60 | 0.74 |  |
| 23 | Perception: CBT too complicated (R) | 0.78 | 0.79 |  |
| 24 | Perception: CBT appropriate for diverse students | 0.82 | 0.85 |  |
|  | **Scale 4: Knowledge** |  |  | 0.58 |
| 25 | CBT diagram | 0.31 | 0.82 |  |
| 26 | Damien: Exposure selection | 0.74 | 0.47 |  |
| 27 | Damien: Cycle of avoidance | 0.46 | 0.22 |  |
| 28 | Dana: Exposure mechanics | 0.21 | 0.49 |  |
| 29 | Dana: Exposure selection | 0.51 | 0.53 |  |
| 30 | Anthony: Behavioral activation | 0.45 | 0.41 |  |
| 31 | Anthony: Psychoeducation - feelings | 0.56 | 0.24 |  |
| 32 | Anthony: Psychoeducation - rationale | 0.54 | 0.50 |  |
| 33 | Jessica: Relaxation | 0.54 | 0.13 |  |
|  | **CCS Total** |  |  | 0.92 |

**SCORING THE SCALES**

***Scale 1. Non-behavioral Skills (N=10)***

Compute a mean score for the 10 items that load on this subscale. Items 1-4, 9-14 constitute Scale 1. Ranges from 0 to 4.

***Scale 2. Behavioral Skills (N=8)***

Compute a mean score for the 8 items that load on this subscale. Items 5-8, 15-18 constitute Scale 2. Ranges from 0 to 4.

***Scale 3. Perceptions (N=6)***

First, create binary responses for the items that load on this subscale, Scale 3 (items 19-24), following the instructions below. The items 21 and 23 must be reverse scored. 1 indicates positive and 0 indicates non-positive perceptions of CBT.

- For items 19, 20, 22, 24: “agree” = 1; “disagree” = 0; “don’t know” = 0
- For items 21, 23: “disagree” = 1; “agree” = 0; “don’t know” =0

Second, compute a mean score for the 6 binary items, and multiply by 4. Ranges from 0 to 4.

***Scale 4. Knowledge (N=9)***

First, grade answers for the items that load on this subscale, Scale 4 (items 25-33). 1 indicates correct answers and 0 indicates incorrect answers. See below for the answer key.

Second, compute a mean score for the 9 graded items, and multiply by 4. Ranges from 0 to 4.

| Item # | Item | *Correct answer* |
| --- | --- | --- |
| 25 | CBT diagram | All three must be correct:  1=Thoughts (thought, thinking, beliefs, cognitions, interpretation, automatic though, activating thought, think)  2=Feelings (feeling, emotion, emotions, mood)  3=Behaviors (behavior, action, actions, choice, choices, doing, behavior response, behave) |
| 26 | Damien: One exposure that you might have Damien do would be: | Ask him to raise his hand in class |
| 27 | Damien: Cycle of avoid | Avoidance feels good in the short term |
| 28 | Dana: Exposure | True |
| 29 | Dana: Exposure target | Making an intentional mistake on her homework |
| 30 | Anthony: Behavioral activation | Behavioral Activation |
| 31 | Anthony: Feelings thermometer | Feeling down is different from feeling devastated |
| 32 | Anthony: Psychoeducation | Normalize his symptoms and engage him in treatment |
| 33 | Jessica: Relaxation | Relaxation may help with sleep difficulties that are common in depression |

**COMPUTING THE TOTAL SCORE**

Follow instructions above to calculate the subscale scores. It is recommended not calculating the subscale score if a respondent was missing more than half of the subscale items. Compute a mean of the subscale scores for the total score. Ranges from 0 to 4. It is recommended not calculating the total score if as respondent was missing two or more subscale scores.
